# Identification of Eupatilin from *Artemisia argyi* as a Selective PPARα Agonist Using Affinity Selection Ultrafiltration LC-MS

**DOI:** 10.3390/molecules200813753

**Published:** 2015-07-28

**Authors:** Yongsoo Choi, Yujung Jung, Su-Nam Kim

**Affiliations:** 1 Laboratory of Biomodulation, Natural Products Research Institute, Korea Institute of Science and Technology, Gangneung 210-340, Korea; E-Mail: yongsoo.choi@kist.re.kr; 2 Laboratory of Natural Skinomics, Natural Products Research Institute, Korea Institute of Science and Technology, 290 Daejeon-dong, Gangneung-si, Gangwon-do 210-340, Korea; E-Mail: yj0103@kist.re.kr

**Keywords:** PPARα, eupatilin, *Artemisia argyi*, ultrafiltration LC-MS

## Abstract

Peroxisome proliferator-activated receptors (PPARs) are key nuclear receptors and therapeutic targets for the treatment of metabolic diseases through the regulation of insulin resistance, diabetes, and dyslipidemia. Although a few drugs that target PPARs have been approved, more diverse and novel PPAR ligands are necessary to improve the safety and efficacy of available drugs. To expedite the search for new natural agonists of PPARs, we developed a screening assay based on ultrafiltration liquid chromatography-mass spectrometry (LC-MS) that is compatible with complex samples such as dietary foods or botanical extracts. The known PPARα and/or PPARγ ligands resveratrol and rosiglitazone were used as positive controls to validate the developed method. When applied to the screening of an *Artemisia argyi* extract, eupatilin was identified as a selective PPARα ligand. A PPAR competitive binding assay based on FRET detection also confirmed eupatilin as a selective PPARα agonist exhibiting a binding affinity of 1.18 μM (IC_50_). Furthermore, eupatilin activation of the transcriptional activity of PPARα was confirmed using a cell-based transactivation assay. Thus, ultrafiltration LC-MS is a suitable assay for the identification of PPAR ligands in complex matrixes such as extracts of dietary foods and botanicals.

## 1. Introduction

The peroxisome proliferator-activated receptors (PPARs) are members of the nuclear hormone receptor superfamily of ligand-activated transcription factors and are currently appreciated as potential therapeutic targets for the treatment of diabetes and dyslipidemia. Among the three isoforms of PPARs (PPARα, PPARβ/δ, and PPARγ), PPARα plays an important role in the regulation of fatty acid oxidation and lipid metabolism and is beneficial for its protection against metabolic disorders associated with type 2 diabetes and obesity. [1] PPARα is a key regulator of lipid metabolism and is highly expressed in the liver, muscle, heart, and kidney, and pharmacological activation of this receptor results in anti-inflammatory activities in the liver and in adipose and vascular tissues. PPARα stimulates lipid consumption by enhancing the expression of fatty acid oxidation genes, resulting in the amelioration of hyperlipidemia [2].

In parallel with the important biological roles of PPARα, PPARγ has also been recognized as a therapeutic target to treat hyperglycemia associated with metabolic syndrome and type 2 diabetes [3]. As observed for PPARα, PPARγ also regulates genes related to lipid metabolism, immunity, and inflammation [4]. Although a few drugs that target PPARγ, such as troglitazone, rosiglitazone, and pioglitazone, have been approved for patients with type 2 diabetes, severe adverse effects have led to interest in the discovery of more diverse and novel compounds that target PPARα or PPARγ.

Natural products have been an important source for the discovery and development of new agents to treat many diseases, and numerous natural product ligands that target PPARγ have been identified, whereas only a few natural agonists of PPARα have been reported to date. The identification of novel agonists of PPARα or PPARγ from complex extracts of dietary foods and botanicals requires a sensitive and selective screening assay to increase throughput and to reduce the incidence of false positives. Although sensitive in sensing a binding interaction between a compound and a receptor, methods based on spectroscopic detection such as fluorescence [5,6,7] and surface plasmon resonance [8,9,10] require a probe tag or chemical immobilization of the receptor and do not provide direct structural information about the positive compounds.

To facilitate the discovery of novel PPAR ligands, with a particular interest in identifying natural PPARα agonists, an affinity-based mass spectrometry screening assay using ultrafiltration was developed in this study. When a target protein or enzyme is soluble, this ultrafiltration liquid chromatography-mass spectrometry (LC-MS) assay can be particularly useful because the target receptor is maintained in solution during binding and screening, and a chemical tagging or immobilization step is not necessary [11].

Previous studies have indicated that an ultrafiltration LC-MS assay is suitable to screen for natural product ligands for nuclear receptors such as estrogen receptors [12,13] and retinoid X receptors [14], as well as the cytosolic enzyme quinone reductase 2 [11]. Using a similar principle, an ultrafiltration LC-MS assay was developed in this study to identify PPAR ligands, resulting in the identification of a ligand from an *Artemisia* species extract that was found to be the strongest natural agonist of PPARα identified to date. Finally, a TR-FRET PPAR competitive binding assay and a cell-based transactivation assay were performed to determine the binding affinity of this PPARα agonist and to investigate its transactivation activity in cells.

## 2. Results and Discussion

### 2.1. Validation of the Ultrafiltration LC-MS Assay for PPAR Screening

Ultrafiltration LC-MS screening has been demonstrated to be useful when used with soluble molecular targets, such as nuclear receptors, because the target is maintained in solution during binding and screening. Ultrafiltration LC-MS has been successfully used to identify bioactive ligands for estrogen receptors [12,13], retinoid X receptors [14], and the quinone reductase 2 cytosolic enzyme [11]. In particular, the screening of complex natural products and the characterization of each ligand can be facilitated when ultrafiltration is combined with LC-MS and tandem mass spectrometry with high-resolution accurate mass measurements by directly providing tentative structural information about the ligand without further subfractionation. Because PPAR receptors are also a type of nuclear receptor and play important biological roles in metabolic diseases, a solution-phase screening technique using ultrafiltration LC-MS was developed to address the unmet need for the discovery of selective PPAR ligands from natural products such as medicinal herbs and foods.

To verify that the ultrafiltration LC-MS assay could detect PPARα and PPARγ ligands, the known ligands rosiglitazone and resveratrol were selected for the validation screening. Rosiglitazone is known to be a selective PPARγ agonist and exhibits a binding affinity of 80 nM [15], whereas resveratrol was found to bind to both ligand-binding domains of PPARα and PPARγ, with binding affinities of 2.7 μM and 1.4 μM, respectively [16]. Although numerous agonists of PPARγ have been identified from dietary foods and herbs [17,18,19,20,21], only a few natural agonists of PPARα, such as resveratrol, have been reported, among which resveratrol exhibits the strongest binding to PPARα to date.

**Figure 1:**
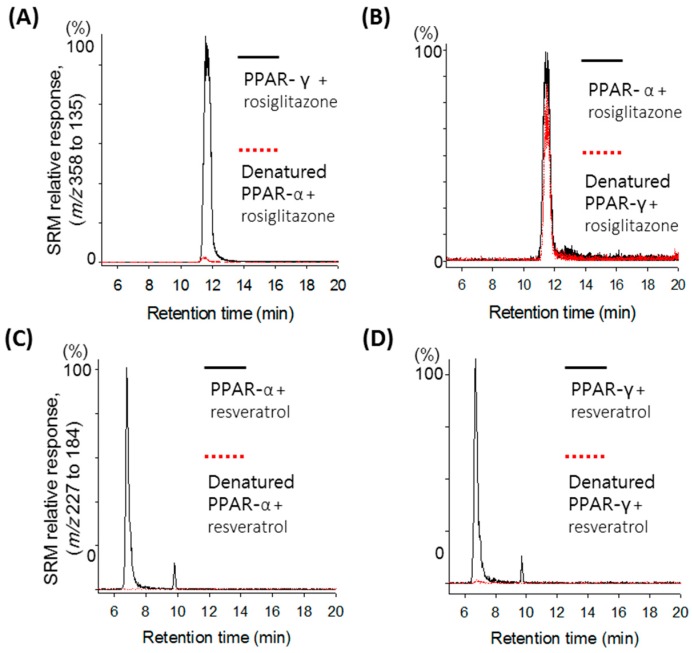
Ultrafiltration LC-MS screening of rosiglitazone and resveratrol for binding to PPARγ and PPARα. Denatured receptor was used as a control for nonspecific binding (dotted line). Enhancement of the rosiglitazone peak compared with the control was observed upon incubation with PPARγ (**A**); but not PPARα (**B**); whereas enhancement of the resveratrol peak was observed with both PPARγ (**C**) and PPARα (**D**).

Using freshly prepared receptor as a positive control and denatured receptor as a negative control, LC-MS/MS analysis of the ultrafiltrates following binding, washing, and release of the ligand from each receptor was performed, and the results are shown in Figure 1. A protonated molecular ion at *m*/*z* 358 and its fragmented ion at *m*/*z* 153 were used to detect rosiglitazone, whereas a deprotonated molecular ion at *m*/*z* 227 and its fragmented ion at *m*/*z* 185 were used to detect resveratrol.

As shown in Figure 1A, a strong rosiglitazone signal at a retention time of 11.92 min in the positive control containing fresh PPARγ was detected, whereas a weak signal in the negative control containing denatured PPARγ was detected due to nonspecific binding of rosiglitazone to the receptor. Significant signal enhancement in the positive control compared with the negative control indicates specific binding of the ligand to the receptor. A signal enhancement for rosiglitazone between the positive and the negative controls was not observed for PPARα, and only a weak signal due to nonspecific binding of rosiglitazone was detected in both the positive and the negative controls (Figure 1B). These results are consistent with the fact that rosiglitazone is a selective PPARγ agonist. For resveratrol, a strong resveratrol signal at 6.81 min was detected in each ultrafiltrate containing PPARγ (Figure 1C) and PPARα (Figure 1D), confirming that resveratrol is a dual agonist of PPARγ and PPARα.

With respect to detection capability of the screening method developed for PPARs at 1 μM, incubation concentration of rosiglitazone could be lowered to be 0.2 μM using the Q-TOF MS instrument and ten times lower incubation concentration could be used under the same conditions when the tandem mass spectrometry was used. Thus, for a primary PPAR ligand screening of large number of natural extracts, competitive incubation of a positive compound with test extract using tandem mass spectrometry can be more useful method with respect to screening time and purchase cost of the receptor.

### 2.2. Screening of the A. argyi Extract Using the Ultrafiltration LC-MS Assay

**Figure 2:**
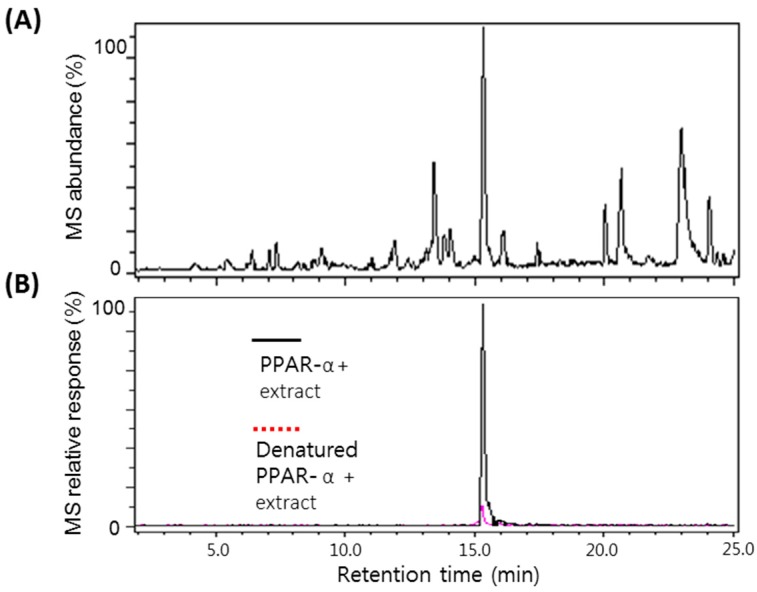
LC-MS chromatograms of an *Artemisia argyi* methanol extract (**A**) and detection of a positive compound (**B**) as a PPARα ligand using ultrafiltration LC-MS. MetaboliteDetect at a setting of greater than threefold enhanced signal and less than 0.05 *m*/*z* difference automatically detected a positive compound in the LC-MS chromatograms of the extract.

**Figure 3:**
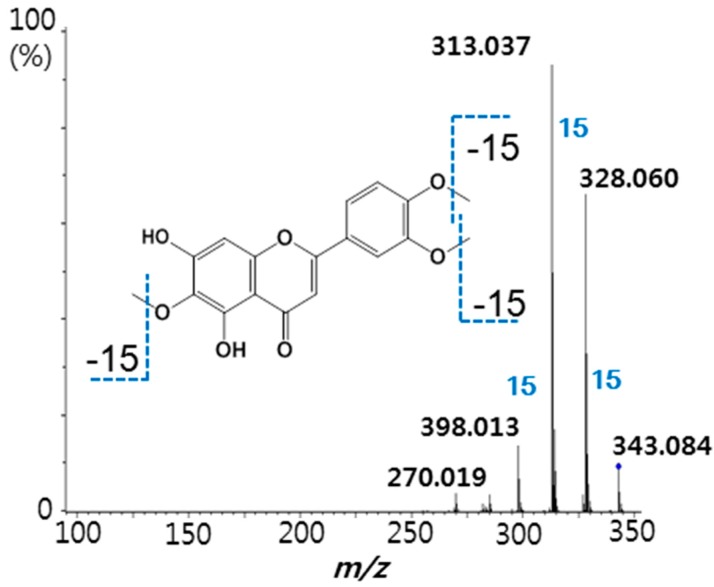
Negative ion electrospray tandem mass spectra of the deprotonated molecule of eupatilin at *m*/*z* 343. Product ion mass spectra were obtained using collision-induced dissociation at 18 eV energy.

Following confirmation that ultrafiltration LC-MS/MS could be used to selectively detect PPARα ligands, this assay was applied to screen a series of natural product extracts. Figure 2A shows a representative LC-MS chromatogram of the *A. argyi* extract. As shown in the ultrafiltration LC-MS chromatograms in Figure 2B, a positive compound was detected in an *A. argyi* extract that eluted at 15.21 min with a deprotonated molecule of *m*/*z* 353. Enhancement of this peak greater by more than 5-fold compared with the control containing denatured receptor indicated specific binding of the ligand to PPARα. From the determination of the accurate molecular mass of the ligand in the extract, the ligand could be tentatively identified as eupatilin, and a eupatilin standard was analyzed for comparison with the extract sample. Based on the exact molecular weight, identical MS/MS fragmentation patterns, and coelution with an authentic standard during LC-MS, the ligand in the extract was confirmed as eupatilin and determined to be contained 1.6% in it. Representative fragmented ion mass chromatograms of eupatilin at 18 eV collision energy are shown in Figure 3. The fragment ions of *m*/*z* 338 and *m*/*z* 323 correspond to the loss of one and two methyl groups of the molecular ion, respectively, and this fragmentation pattern has been reported to be a typical characteristic for methylated flavonoids [22].

*Artemisia* species (Compositae) have been used in traditional medicine for the treatment of various carcinomas [23,24,25], scleromas, indurations of various organs, inflammation [26,27,28] and other disorders. *Artemisia* species represent potential sources of novel lead compounds to treat diabetes [27,29,30]. Artemisinin and its derivatives have been reported to exhibit relatively low toxicity in humans, whereas these compounds are cytotoxic to human cancer cells. Therefore, *Artemisia* species may exert anti-inflammatory effects and protect against metabolic disorders in part via the direct interaction of eupatilin with PPARα.

### 2.3. Binding Affinity of Eupatilin and Functional Cell-Based Assay

**Figure 4:**
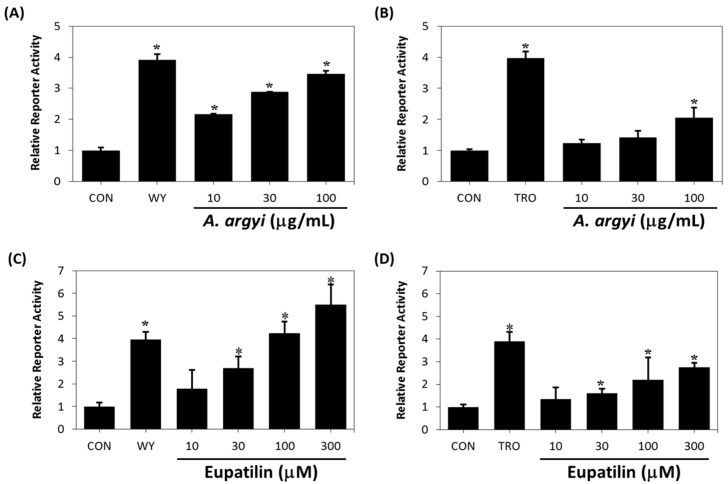
The *A. argyi* extract and eupatilin activate PPARα (**A**,**C**) and PPARγ (**B**,**D**) *in vitro*. Human PPARα and PPARγ expression vector, PPRE-luciferase reporter construct, and pRL-SV40 vector were transiently cotransfected in CV-1 cells. The cells were then treated with 10 μM WY14643 (WY), 10 μM troglitazone (TRO) or various concentrations of *A. argyi* extract (10, 30, 100 μg∙mL^−1^) for 24 h. A reporter assay was performed as described in the Experimental Section. Each bar represent the mean ± SD of triplicate experiments. * *p* < 0.05 *vs.* control.

Although effective in the initial screening of natural plant extracts to identify active ligands of the molecular target, the ultrafiltration LC-MS assay does not determine how well a ligand may function as an agonist. To determine the pharmacological properties of eupatilin, we examined the effects of the *A. argyi* extract and eupatilin on transcriptional activation of PPARs. As shown in Figure 4A,C, the *A. argyi* extract and eupatilin treatment led to an increase in PPARα-reporter gene activities in a dose-dependent manner and the EC_50_ values to induce PPARα activation were estimated to be 10.8 μg∙mL^−1^ and 41.9 μM, respectively. However, the *A. argyi* extract and eupatilin minimally affected PPARγ (Figure 4B,D), and no detectable effect on the transcriptional activation of PPARδ and RXRα was observed (data not shown). We next explored the binding affinities of eupatilin to PPARα and PPARγ, using a competitive binding assay based on TR-FRET and found that eupatilin could bind to PPARα, not PPARγ and the binding affinity of eupatilin for PPARα was 1.18 μM (Table 1). Previously we reported that PPARα/γ dual agonist amorphastilbol bind to PPARα with binding affinity of 1.5 μM and transactivate it with EC_50_ of 7.4 μM [31].

Because of cell permeability of compound, the concentration of activation for PPARs in cells is higher than the concentration of direct binding for PPARs. This is well accordance with the ability of eupatilin to bind to and activate PPARα. Our findings suggest that eupatilin acts as a selective PPARα agonist through direct binding to the PPARα ligand-binding domain.

**Table 1:** Binding affinities of eupatilin for PPARα and PPARγ.

Compound	IC_50_	
	PPARα	PPARγ
GW6471	226.9 nM	-
GW9662	-	24.3 nM
Eupatilin	1.18 μM	>100 μM

## 3. Experimental Section

### 3.1. Chemicals and Reagents

Standard chemicals, including *trans*-resveratrol, rosiglitazone, and GW9662 were purchased from Sigma-Aldrich (St. Louis, MO, USA); eupatilin was purchased from AdipoGen (San Diego, CA, USA); and all solvents were of HPLC grade and obtained from Fisher Scientific (Hanover Park, IL, USA). Centrifugal ultrafiltration filters (Microcon YM-10) were purchased from Millipore (Bedford, MA, USA). Human recombinant ligand-binding domains of PPARα and PPARγ were obtained from Cayman Chemical (Ann Arbor, MI, USA). The *A. argyi* (Compositae) was purchased from a local market (Kyungdong Herb-Market, Seoul, Korea) and authenticated by K.H. Kim (Sungkyunkwan University). A voucher specimen (KIST-089) has been deposited in the herbarium of the KIST Gangneung Institute, Gangneung, Korea. An *A. argyi* extract was prepared from 100% methanol with sonication for 10 min.

### 3.2. Binding to PPARα or PPARγ and Ultrafiltration

For ultrafiltration LC-MS screening, the natural product extract (100 μg∙mL^−1^), rosiglitazone (10 μg∙mL^−1^), or resveratrol (50 μg∙mL^−1^) was incubated with 5 μM receptor in 100 μL of Tris buffer consisting of 10% glycerol, 50 mM KCl, 1 mM EDTA, and 0.1 mM DTT at room temperature for 2 h. Following incubation, each sample was filtered through a 10,000 Da molecular weight cutoff ultrafiltration membrane by centrifugation at 13,000 *g* for 8 min at 4 °C. The receptor-ligand complexes were washed three times with 150-μL aliquots of 50 mM ammonium acetate (pH 8.0), followed by an additional centrifugation at 13,000 *g* for 12 min to remove the unbound compounds. The washed solution was transferred to a new 10,000 Da molecular weight cutoff ultrafiltration centrifuge tube, and the ligands were dissociated from the receptor using 400 μL of methanol. Following centrifugation, the ultrafiltrate containing the ligands was dried using a SpeedVac system (Thermo Savant SPD1010, Holbrook, NY, USA). Following reconstitution in 100 μL of 50% aqueous methanol, the ligands were analyzed using LC-MS/MS or LC-HR Q-TOF. Identical incubations using denatured receptor were used to control for nonspecific binding.

### 3.3. LC-MS and LC-MS/MS

Each ultrafiltrate was analyzed using a triple quadrupole mass spectrometer (AB SCIEX API 4000 QTRAP^®^, Foster City, CA, USA) or a high-resolution Q-TOF mass spectrometer (Bruker Micromass II, Karlsruhe, Germany). The triple quadrupole mass spectrometer was interfaced to an Agilent HPLC system (HP1200, Palo Alto, CA, USA) and the Q-TOF mass spectrometer was interfaced to an Agilent 1260 HPLC system. For LC-MS/MS or Q-TOF MS analysis of ligand-containing ultrafiltrates, a 22 min linear gradient was used from 15% to 70% aqueous acetonitrile, and 10 μL of each sample was directly injected onto a Kinetex C_18_ column (Phenomenex; 100 mm × 2.1 mm, 2.6 μm) for chromatographic separation. All flow rates were 200 μL∙min^−1^, and the column was re-equilibrated at least 10 min between analyses. For LC-MS/MS analysis, the protonated molecule of rosiglitazone was ionized by 5.5 kV of ESI (+) voltage, and the deprotonated molecule of resveratrol or eupatilin was ionized by −4.5 kV of ESI (−) voltage. The probe temperature was set to 400 °C. Nitrogen was used as the curtain gas and as ion source gas 1 and 2 at settings of 35, 30, and 50, respectively. Selective monitoring parameters for each compound were set as described in Table 2, and dwell times were set to 200 ms for each transition. High-resolution accurate mass measurements of the *A. argyi* extract were acquired for screening using the Q-TOF mass spectrometer over the range *m*/*z* 150 to *m*/*z* 800 every 0.5 s. Tandem mass spectra of eupatilin were acquired at 18 eV of collision energy for collision-induced dissociation, and ion source parameters included a capillary voltage of −4.0 kV, a source block temperature of 180 °C, a nebulizer pressure of 0.8 bar, and a dry gas flow rate of 8.0 L∙min^−1^.

**Table 2:** LC-MS/MS parameters to detect resveratrol and rosiglitazone.

Compound	SRM Transition (*m*/*z*)	DP	EP	CE	CXP
Resveratrol	227 to 185	−105	−10	−26	−9
Rosiglitazone	358 to 135	116	10	39	8

### 3.4. Cell-Based Transactivation Assay

CV-1 cells were seeded into 24-well plates and cultured for 24 h before transfection. Prior to transfection, the medium was replaced with 10% charcoal dextran-treated FBS–DMEM. After 4 h, a DNA mixture containing a 3× multimerized PPRE-luciferase reporter plasmid (0.3 μg) and the internal control plasmid pRL-SV40 (5 ng) were transfected using the TransFast™ transfection reagent (Promega, Madison, WI, USA). Twenty-four hours after transfection, the cells were treated with 10 μM WY-14643 or the indicated concentrations of eupatilin or *A. argyi* extract and were incubated for an additional 24 h. The luciferase activities of the cell lysates were determined using the Dual-Luciferase^®^ Reporter Assay System according to the manufacturer’s instructions (Promega). The relative luciferase activity was normalized to the corresponding *Renilla* luciferase activity to determine the transfection efficiency.

### 3.5. Ligand Binding Assay

The LanthaScreen™ TR-FRET PPAR competitive binding assay (Invitrogen, Carlsbad, CA, USA) was performed according to the manufacturer’s instructions. A mixture of 5 ng of a fusion protein consisting of glutathione-*S*-transferase and the PPAR ligand-binding domain (GST-PPAR-LBD), 5 nM Tb-GST antibody, 5 nM Fluormone™ Pan-PPAR Green, and serial dilutions of eupatilin ≤ 100 μM were added to the wells of black 384-well low-volume 21 plates (Greiner Bio-One GmbH, Kremsmünster, Austria) for a total volume of 18 μL. All of the dilutions were prepared using TR-FRET assay buffer C. The experiments were performed in triplicate, and the samples were incubated for 2 h in the dark prior to analysis using an Infinite^®^ M1000 microplate reader (TECAN). The FRET signal was measured by excitation at 340 nm and emission at 520 nm for fluorescein and 490 nm for terbium. The TR-FRET ratio was calculated using the 520 nm/490 nm ratio. A competition curve was generated by plotting the TR-FRET ratio *vs.* the log [test compound]. To determine the IC_50_ value, the data were fit using an equation for a sigmoidal dose-response.

### 3.6. Statistical Analyses

The data are expressed as the means ± SD. Differences between the mean values in the two groups were analyzed using one-way analysis of variance (ANOVA). *p* < 0.05 was considered statistically significant.

## 4. Conclusions

In conclusion, an ultrafiltration LC-MS assay developed in this study resulted in the identification of a natural PPARα agonist, although the applicability of this technique is not limited to this receptor alone. Screening of a series of natural plants using this method identified eupatilin in an *A. argyi* extract as a selective PPARα agonist and represents the first PPARα agonist identified from the *A. argyi*. To the best of our knowledge, eupatilin may represent the most potent PPARα agonist among natural product agonists identified to date.
